# Ebola Virus Uptake into Polarized Cells from the Apical Surface

**DOI:** 10.3390/v11121117

**Published:** 2019-12-02

**Authors:** Meng Hu, Fei Wang, Wei Li, Xiaowei Zhang, Zhiping Zhang, Xian-En Zhang, Zongqiang Cui

**Affiliations:** 1 State Key Laboratory of Virology, Wuhan Institute of Virology, Center for Biosafety Mega-Science, Chinese Academy of Sciences, Wuhan 430071, China; damonhu@whu.edu.cn (M.H.); wangfei31415927@126.com (F.W.); liwei@wh.iov.cn (W.L.); zhangxw@wh.iov.cn (X.Z.); zhangzp@wh.iov.cn (Z.Z.); 2 University of Chinese Academy of Sciences, Beijing 100049, China; 3 National Laboratory of Biomacromolecules, Institute of Biophysics, Chinese Academy of Sciences, Beijing 100101, China; zhangxe@ibp.ac.cn

**Keywords:** Ebola virus, polarized cells, apical entry, TIM-1, Axl

## Abstract

Ebola virus (EBOV) causes severe hemorrhagic fever with high mortality rates. EBOV can infect many types of cells. During severe EBOV infection, polarized epithelial and endothelial cells are damaged, which promotes vascular instability and dysregulation. However, the mechanism causing these symptoms is largely unknown. Here, we studied virus infection in polarized Vero C1008 cells grown on semipermeable Transwell by using EGFP-labeled Ebola virus-like particles (VLPs). Our results showed that Ebola VLPs preferred to enter polarized Vero cells from the apical cell surface. Furthermore, we showed that the EBOV receptors TIM-1 and Axl were distributed apically, which could be responsible for mediating efficient apical viral entry. Macropinocytosis and intracellular receptor Niemann–Pick type C1 (NPC1) had no polarized distribution, although they played roles in virus entry. This study provides a new view of EBOV uptake and cell polarization, which facilitates a further understanding of EBOV infection and pathogenesis.

## 1. Introduction

The filovirus Ebola virus (EBOV) is an enveloped, negative-sense, single-stranded RNA virus [1,2,3]. The *Ebolavirus* genus contains five species, *Bundibugyo ebolavirus*, *Reston ebolavirus*, *Sudan ebolavirus*, *Tai Forest ebolavirus*, and *Zaire ebolavirus*. Members of all species can infect humans, but Reston virus is the only member of the ebolavirus species that seems to be avirulent in humans [4,5]. Recently, a number of severe hemorrhagic fever outbreaks with mortality rates of up to 90% were caused by EBOV in Africa [6,7]. So far, there is still a lack of available vaccines or antiviral therapies against EBOV.

The EBOV genome encodes seven proteins that include transmembrane glycoprotein (GP), nucleoprotein (NP), VP24, VP30, VP35, VP40, and L protein [8,9]. Expression of VP40 protein alone is sufficient for assembly and formation of virus-like particles (VLPs) that are similar in size and shape [10,11] and nearly indistinguishable from the authentic virion [12]. It has been well documented that Ebola VLPs have the same capability to infect cells as EBOV. EBOV GP, a prototypic class I viral fusion protein, is the only viral surface protein and exists as a homotrimer [13]. During transit to cell surface, GP can be cleaved into GP1 and GP2 subunits by furin-like proteases [14,15]. GP1 is a receptor binding subunit, and GP2 subunit is mainly responsible for the fusion event between the virus and host membrane [16,17]. GP1 and GP2 are disulfide linked and form the mature GP_1,2_ complex. The GP_1,2_ complex mediates virus entry, and it is cleaved into GP_CL_ by cathepsins B and L in the endosomal compartment [18]. The GP_CL_ subunit binds to the intracellular receptor Niemann–Pick type C1 (NPC1) and induces membrane fusion for virus genome release [19,20,21]. TIM-1 (the receptor T-cell immunoglobulin and mucin domain-1) and related phosphatidylserine-binding proteins, such as Axl (a receptor tyrosine kinase), have been shown to promote infection by specific receptor recognition [22,23].

It has been reported that EBOV can infect a wide range of host cells, such as monocytes, macrophages, epithelial cells, and endothelial cells [7,24]. Epithelial and endothelial cells, which have a polarized phenotype and barrier function, are distinctive from most other cell types due to their apical and basolateral plasma membrane domains, which are strictly separated by tight junctions [25,26]. These polarized cells line the cavities of major organs and form selective barriers against the invasion of many pathogens [27]. The membranes of polarized cells are exposed to different physiological environments: the apical membrane faces the lumen, and the basolateral membrane abuts the underlying stratum [28,29]. Although it is important for the pathogenesis of EBOV infection, the virus entry mechanism in polarized cells remains to be determined.

In the present study, we characterized virus internalization in polarized Vero cells grown on semipermeable Transwell by using EGFP-labeled filamentous Ebola virus-like particles (VLPs) consisting of matrix protein VP40 and GP. EBOV VLPs were found to enter polarized cells from the apical cell surface. The EBOV receptors TIM-1 and Axl were found to be apically distributed, which could be responsible for mediating the efficient apical entry of Ebola virus.

## 2. Materials and Methods

### 2.1. Cell Culture

Vero C1008 (obtained from ATCC, CRL-1587) and 293T (obtained from ATCC, ACS-4500) cells were maintained in Dulbecco’s modified Eagle’s medium (DMEM) containing 10% fetal bovine serum (ThermoFisher, Waltham, MA, USA), 100 U/mL penicillin, and 0.1 mg/mL streptomycin at 37 °C under 5% CO2 in a humidified incubator. For the analysis of the polarized cells, Vero C1008 cells were seeded on Transwell filter membranes (Falcon/Corning, Corning, NY, USA) with a 1.0 μm pore size. The culture medium was replaced, and the resistance value was monitored daily using a Millicell ERS-2 Electrical Resistance System (Millipore Corp, Burlington, MA, USA) until polarization was established. At day 5 post-seeding, the cells with a resistance of 60 (±10) Ω were prepared for the following study.

### 2.2. Plasmid and Antibodies

For the construction of EGFP linked with Zaire EBOV VLPs, the EGFP gene was fused with the N terminus of EBOV VP40 (pEGFP-EBOV-VP40). The following antibodies were used in this study: rabbit anti-VP40 IgG (our laboratory), anti-ZO-1 (Thermo Fisher, 1A12), anti-claudin 1 (ab15098), anti-TIM1 (IF: AF1750, R&D; Block function: BioLegend, San Diego, CA, USA, #353902), anti-Axl (Cell Signaling Technology, Danvers, MA, USA, #8661), anti-NPC1 (ab134113), anti-SNX5 (ab5983), anti-EGFP (ab184601), anti-heparan sulfate (ab23418), Alexa Fluor 488- or 555-labeled goat anti-mouse or anti-rabbit IgG (Cell Signaling Technology), Alexa Fluor 647-labeled goat anti-mouse or anti-rabbit IgG (ab150115 or ab150079), Alexa Fluor 594-labeled donkey anti-goat IgG (ab150132), and mouse or rabbit IgG isotype control (Thermo Fisher).

### 2.3. Immunofluorescence Assay

Cells were fixed with 4% paraformaldehyde for 30 min at room temperature, incubated with 0.5% Triton X-100 for 15 min, and blocked with 10% FBS/PBS for 1 h. Cells were incubated with primary antibodies at 4 °C overnight. Then, the cells were washed with 0.1% Tween 20 in PBS three times, followed by incubation with the appropriate fluorescently labeled secondary antibodies for 1 h at 37 °C. After the cells were washed with 0.1% Tween 20 in PBS three times, Hoechst 33342 was used to stain the nucleus for 5 min before observation. The imaging was conducted using a Nikon TiE inverted microscope equipped with a 60×, 1.49 NA, oil immersion objective lens (NIKON).

### 2.4. Production and Purification of VLPs

The plasmids pEGFP-EBOV-VP40 and Zaire EBOV GP were cotransfected into 293T cells grown in 10 cm dishes with 54 μg of total DNA at a ratio of 2:1. Two days after transfection, the supernatants were collected and purified by centrifugation (25,000 rpm, 4 °C, 2.5 h in a Beckman rotor). The viral pellet was resuspended in TNE buffer (10 mM Tris, 100 mM NaCl, and 1 mM EDTA) and dialyzed overnight at 4 °C. The dialysis was performed with membranes of MWCO 8–14 KD (Shanghai Yuanye Bio-Technology, Shanghai, China, MD1425-5m).

### 2.5. SDS-PAGE and Western Blotting

Protein concentrations were determined using the Bradford protein assay kit (Beyotime, Shanghai, China). Equal amounts of protein were separated by 12% SDS-PAGE and transferred to a PVDF membrane. After blocking with 5% nonfat milk in PBST for approximately 2 h, the membrane was incubated with the primary antibodies overnight at 4 °C. After washing with PBST, the membrane was incubated with horseradish peroxidase-conjugated goat anti-mouse or rabbit IgG before imaging.

### 2.6. Flow Cytometry Assay

Cells were washed with 2% FBS/PBS three times and then blocked with 10% FBS/PBS for 20 min. Cells were incubated with blocking antibodies at 37 °C for 30 min. Then, EBOV VLPs were added, and cell cultures were further incubated at 37 °C for 1.5 h. Polarized cells were washed with 2% FBS/PBS three times to remove the cell surface-attached VLPs (Figure S1), and then cells were detached with trypsin (0.25% trypsin containing 0.02% EDTA). Cells were then resuspended in 2% FBS/PBS, concentrated by centrifugation (1000 rpm, room temperature, 10 min), and analyzed by flow cytometry (LSRFortessa, BD).

### 2.7. EIPA Inhibitor Assay

Vero C1008 cells were seeded in Transwells of 1.0 μm pore size at a density of 1 × 10^5^ cells per well. After polarization was established, the cells were incubated with ethylisopropylamiloride (EIPA, a TRPP3 channel inhibitor) or PBS (control group) at 37 °C for 1 h. VLPs were added and incubated at 37 °C for 1.5 h. The cells were washed with 2% FBS/PBS three times and then used in subsequent experiments.

### 2.8. Receptor Inhibitor Assay

The cells were cultured on permeable filter support until polarization was established. The cells were washed three times and incubated with anti-TIM-1 or anti-Axl primary antibodies at 37 °C for 1 h. Normal mouse or rabbit IgG was used in the control group. After blocking, EBOV VLPs were added, and the cells were incubated for 1.5 h. The cells were washed and collected for subsequent experiments.

### 2.9. Dextran Assay

For dextran uptake experiments, cells were seeded on Transwells to establish polarization. Dextran conjugated with Texas Red (70 kDa, Lysine Fixable; Thermo Fisher, D1864) was diluted in medium containing 0.1% bovine serum albumin (BSA) to 1 mg/mL. Either the apical or basolateral surfaces of the polarized cells were incubated with dextran at 37 °C. After 2 h, the cells were washed three times with PBS to remove residual dextran. The cells were collected for subsequent experiments.

## 3. Results

### 3.1. Ebola VLPs Enter Polarized Epithelial Cells from the Apical Membrane

**Figure 1:**
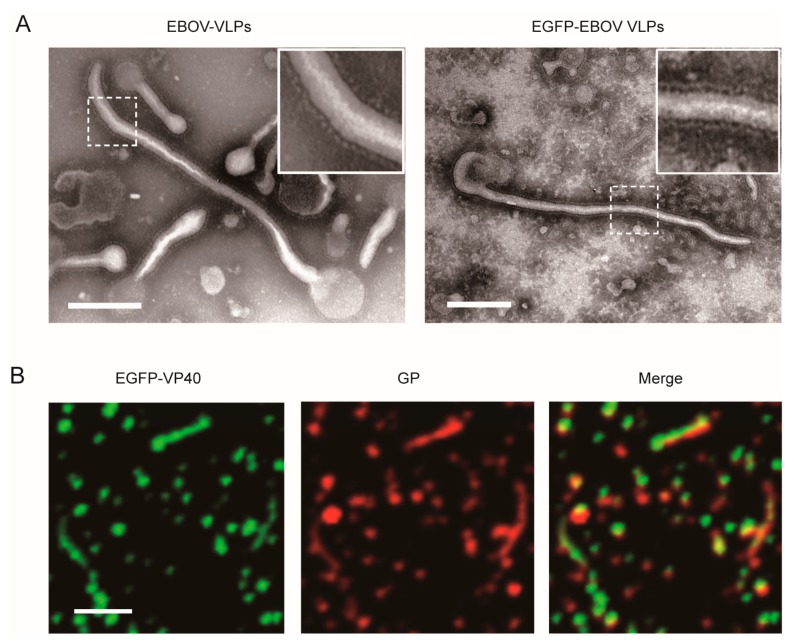
Characterization of Ebola virus-like particles (VLPs) labeled with EGFP. (**A**) TEM images of EGFP-Ebola virus (EBOV) VLPs. Scale bar: 200 nm. (**B**) Fluorescence imaging of the EGFP-labeled VLPs (green: EGFP) that were immunostained with anti-GP antibody (red: Alexa Fluor 555). Scale bar: 1 μm.

To assess the mechanism of EBOV uptake, we established fluorescently labeled Ebola VLPs consisting of matrix proteins VP40 and GP. EGFP was fused to the N terminus of EBOV VP40 (Figure S2A). The expression of target proteins was measured by western blot analysis, and the results demonstrated that EGFP and VP40 were coexpressed (Figure S2B). The EGFP-VLPs were purified and displayed a filamentous-like morphology similar to the wild-type EBOV VLPs visualized by TEM (Figure 1A). Moreover, the EGFP-labeled VLPs were characterized by fluorescence microscopy (Figure 1B). The fluorescence colocalization of EGFP-VP40 and GP indicated that EGFP-VLPs were successfully packaged with GP.

**Figure 2:**
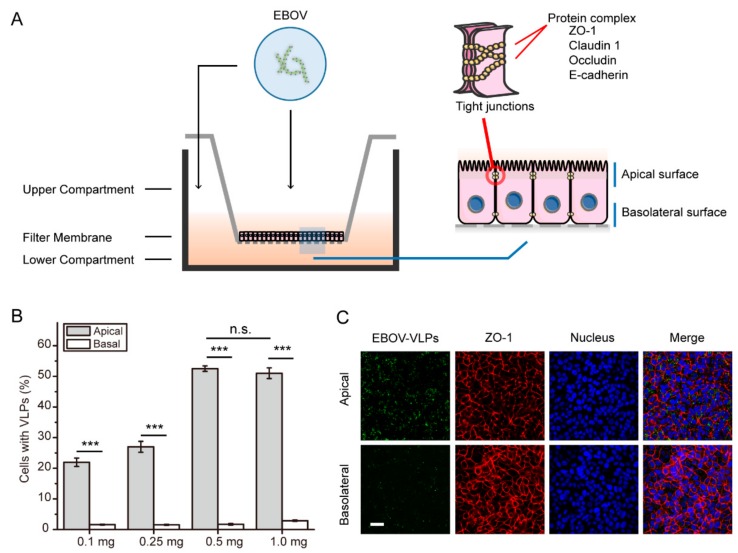
Ebola virus VLPs enter polarized Vero cells from the apical surface. (**A**) Experimental schematic of EBOV entry into polarized cells in the Transwell system. Cells were cultured on Transwell membranes (1.0 μm pore) until polarization was established. EGFP-labeled VLPs were added to either the apical or basolateral chamber and incubated at 37 °C for 1.5 h. Next, the cells were collected and analyzed for subsequent experiments. (**B**) EGFP-EBOV VLPs enter polarized Vero cells from apical surfaces. The percent of infected cells was measured by flow cytometry. (Histograms display averages ± SD; *n* = 3; *** *p* < 0.01; n.s. > 0.05). (**C**) Visualization of the EGFP-EBOV VLPs entering the apical or basolateral membrane. ZO-1: zonula occludens-1. Scale bar: 50 μm.

In previous studies, Vero C1008 cells have been used as a classic model to investigate the interaction between viral pathogens and polarized monolayer cells in vitro [30]. In our work, Vero C1008 cells were grown on the Transwell system until polarization was achieved. In this system, the cells form a polarized, intact monolayer that serves as a mechanical barrier to the virus. Next, the polarized cells were incubated with VLPs either from the apical or from the basolateral domain of the polarized Vero C1008 cells (Figure 2A). A flow cytometry assay was performed to investigate whether EBOV enters Vero cells in a polarized manner. As shown in Figure 2B and Figure S3A,B, the percentage of cells with VLPs peaked when the VLPs were added at 0.5 mg per well (12-well plate). The results showed that the addition of 0.5 mg per well was sufficient for subsequent experiments. The data also demonstrated that the uptake of EBOV VLPs into Vero cells was polarized towards the apical domain. The immunofluorescence results also showed that the amount of EBOV VLPs binding upon apical inoculation of EBOV was higher compared to that upon basolateral inoculation of EBOV in polarized Vero cells (Figure 2B). These data suggest that entry of EBOV into Vero cells is polarized towards the apical domain. In contrast, EBOV VLPs could enter nonpolarized cells equally from every membrane (Figure S4).

### 3.2. Macropinocytosis Is Nonpolarized in Polarized Epithelial Cells

**Figure 3:**
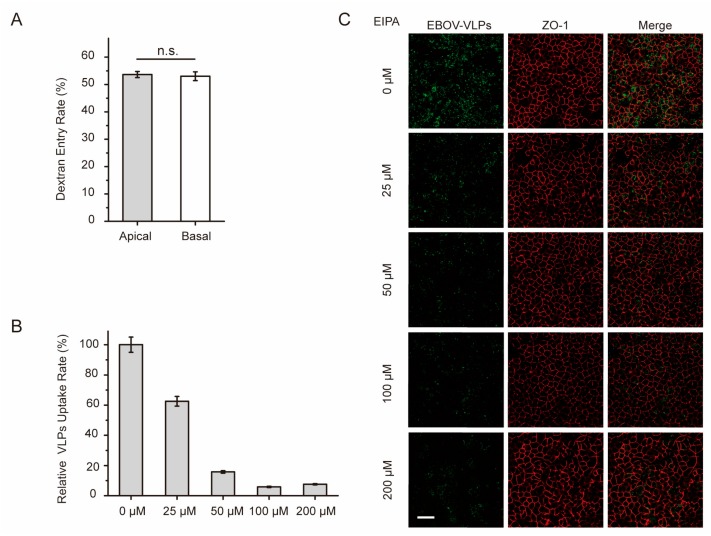
Macropinocytosis is nonpolarized in polarized Vero cells. (**A**) Dextran assay analysis of macropinocytosis in polarized Vero cells grown on a Transwell filter. Either apical or basolateral surfaces of the polarized cells were incubated with dextran conjugated with Texas Red in 37 °C. After 2 h, the cells were analyzed by flow cytometry. Histograms display averages ± SD; *n* = 3; * *p* < 0.05; *** *p* < 0.01; n.s. > 0.05. (**B**) EIPA, an inhibitor of macropinocytosis, obstructs EBOV uptake in a dose-dependent manner. The apical surface of polarized cells was incubated with a gradient concentration of EIPA at 37 °C for 1 h. Then, the cells were incubated with EGFP-VLPs for 1.5 h and analyzed by flow cytometry. Histograms display averages ± SD; *n* = 3; *** *p* < 0.01; n.s. > 0.05. (**C**) Image showing the uptake of EGFP-VLPs in cells incubated with EIPA. Scale bar: 50 μm.

It has been reported that macropinocytosis is the major route for EBOV cell invasion. Thus, we tested whether macropinocytosis was polarized and could be responsible for the polarized uptake of Ebola VLPs. As shown in Figure 3A and Figure S3C, the dextran assay was performed as previously described [31], and the result showed that macropinocytosis could occur equally in both the apical and basolateral domains. The distribution of SNX-5, a marker protein of macropinosomes, was also determined by immunofluorescence assay. As shown in Figure S5, macropinosomes appeared to be evenly distributed in the polarized Vero C1008 cells. In addition, ZO-1 (zonula occludens-1), a tight junction protein, was expressed on cell-to-cell contacts in xy sections and exclusively along lateral plasma membranes of apical domains of adjacent cells in vertical xz sections. These data indicate that macropinocytosis is nonpolarized in polarized cells and should not be the cause of the polarized uptake of Ebola VLPs.

Next, polarized Vero cells were treated with EIPA, a small molecule inhibitor of macropinocytosis, to address the question of whether macropinocytosis is involved in the internalization of Ebola VLPs. As shown in Figure 3B and Figure S3D, EIPA inhibited the uptake of VLPs in polarized Vero cells in a dose-dependent manner. Fluorescence imaging demonstrated that the uptake rate of EBOV VLPs was reduced with increasing doses of EIPA (Figure 3C). These results showed that macropinocytosis was involved in the uptake of Ebola VLPs into polarized Vero cells. 

Overall, it can be speculated that although macropinocytosis participates in EBOV uptake, this process is not responsible for the polarized viral entry from the apical membrane in polarized epithelial cells.

### 3.3. TIM-1 Plays a Role in Apical Domain Binding of EBOV and Efficient Virus Entry in Polarized Vero Cells

**Figure 4:**
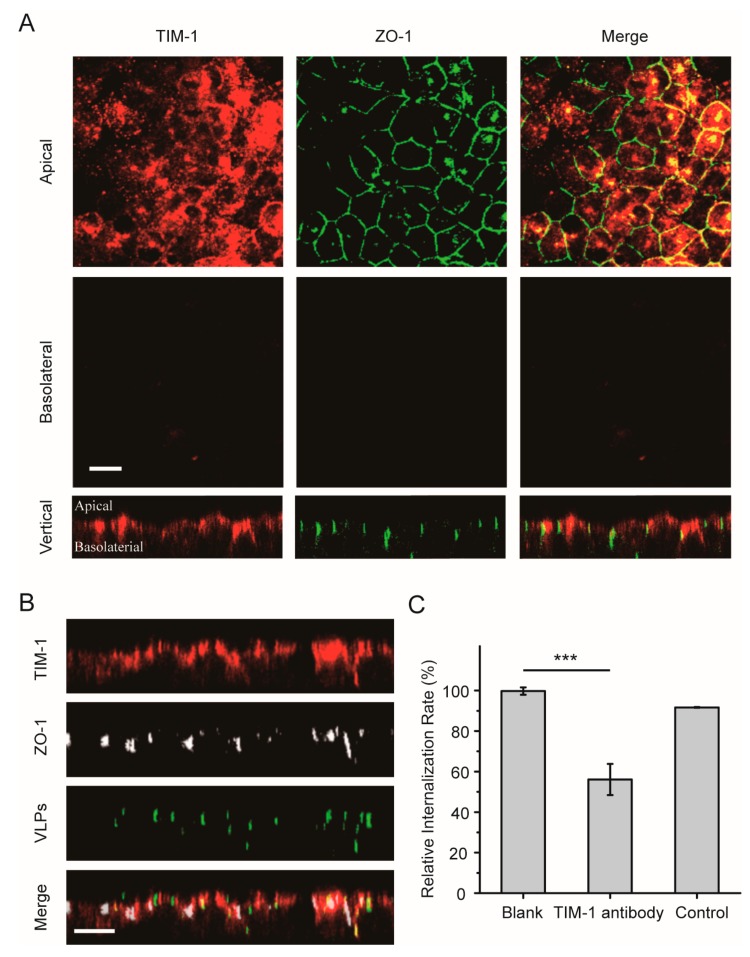
Apical distribution of the TIM-1 receptor in polarized Vero cells. (**A**) TIM-1 staining was detected in apical sections. Polarized Vero cells grown on filter support were fixed with 4% PFA and incubated with anti-TIM-1 antibody (red: Alexa Fluor 594). Tight junctions were stained with an antibody directed against ZO-1 (green: Alexa Fluor 488). Scale bar: 10 μm. (**B**) Confocal images representing the distribution of TIM-1 receptors during EGFP-VLP internalization into polarized Vero cells. The cells were incubated with EGFP-EBOV VLPs for 1.5 h, and then TIM-1 distribution was assessed. Scale bar: 10 μm. (**C**) EGFP-VLP uptake was inhibited by blocking the TIM-1 receptor. Histograms display averages ± SD; *n* = 3; *** *p* < 0.01; n.s. > 0.05.

Next, the role of the EBOV receptor TIM-1 during virus entry was examined in polarized epithelial cells. The intracellular distribution of TIM-1 was first analyzed in polarized Vero cells by using immunofluorescence assay. As shown in Figure 4A, most TIM-1 staining was detected in the apical domain. In vertical xz sections, TIM-1 distribution around the polarized cells could be observed, demonstrating an apical polar localization of the receptor in polarized Vero cells. After the cells were incubated with Ebola VLPs, some TIM-1 molecules were internalized with the VLPs, as shown in Figure 4B. This result is consistent with that in nonpolarized cells (Figure S6A).

To confirm whether the TIM-1 receptor determines the uptake rate in the apical domain, polarized Vero cells were incubated with Ebola VLPs by adding labeled VLPs to the apical filter chamber, and the polarized Vero cells were incubated or not with anti-TIM-1 antibody. After 2 h, the cells were fixed with 4% paraformaldehyde (PFA), and EBOV-positive cells were detected by flow cytometry. As shown in Figure 4C and Figure S3E, positive signals were reduced by more than 40% after the addition of VLPs on the apical sides in the TIM-1-antibody blocked group compared with the blank control group, demonstrating that EBOV utilizes the TIM-1 receptor for entry into polarized epithelial cells. 

### 3.4. The Axl Receptor Is Involved in the Polarized Entry of EBOV

**Figure 5:**
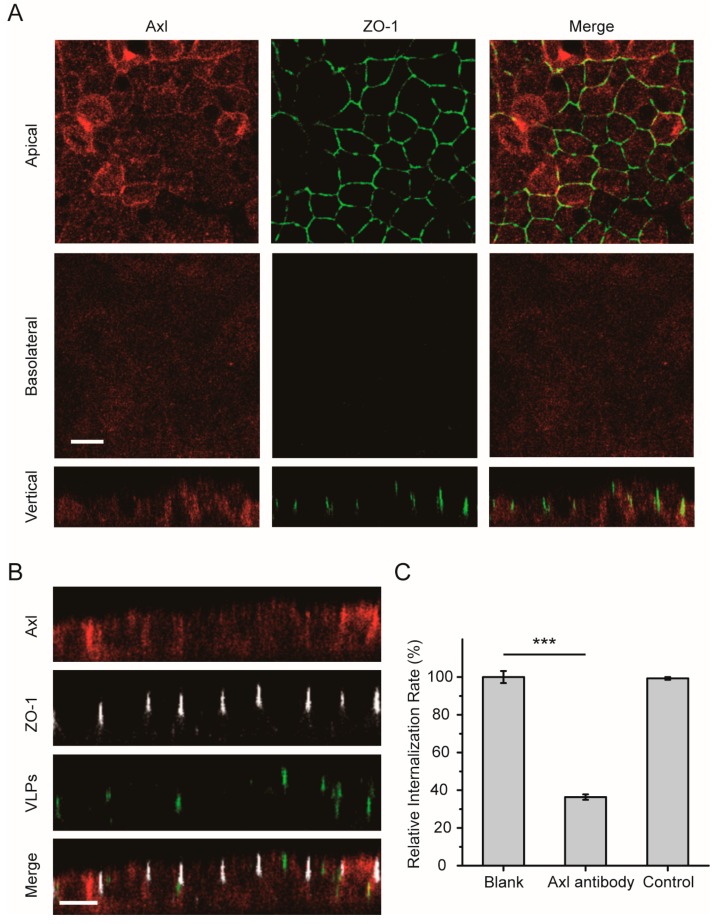
Apical distribution of the Axl receptor in polarized Vero cells. (**A**) Axl appeared to be preferentially apically distributed. Polarized Vero cells were fixed and incubated with anti-Axl antibody (red: Alexa Fluor 555). ZO-1 was stained with anti-ZO-1 antibody (green: Alexa Fluor 488). Scale bar: 10 μm. (**B**) Confocal images representing the distribution of the Axl receptor during EGFP-VLP internalization into polarized Vero cells. Scale bar: 10 μm. (**C**) EGFP-VLP uptake was inhibited by blocking the Axl receptor. Histograms display averages ± SD; *n* = 3; *** *p* < 0.01; n.s. > 0.05.

In addition to the TIM-1 receptor on the surface cell membrane, other receptors for EBOV have been described to date in nonpolarized cells, including Axl and NPC1. We next analyzed the cell surface distribution of the Axl receptor in polarized cells. As shown in Figure 5A, Axl was observed mainly at the apical cell surface membrane, although it was also weakly detected at the basolateral surface. After the cells were incubated with Ebola VLPs, some of the Axl receptors were translocated into the cytoplasm with the Ebola VLPs (Figure 5B). Similar results were observed in nonpolarized cells (Figure S6B). In Figure 5C and Figure S3F, the flow cytometry data showed that less than 50% of the VLP signal could be detected in the cells when endogenous Axl was blocked with a specific antibody. These data suggest that the Axl receptor also participates in polarized EBOV entry.

### 3.5. The NPC1 Receptor Shows No Polarized Distribution

**Figure 6:**
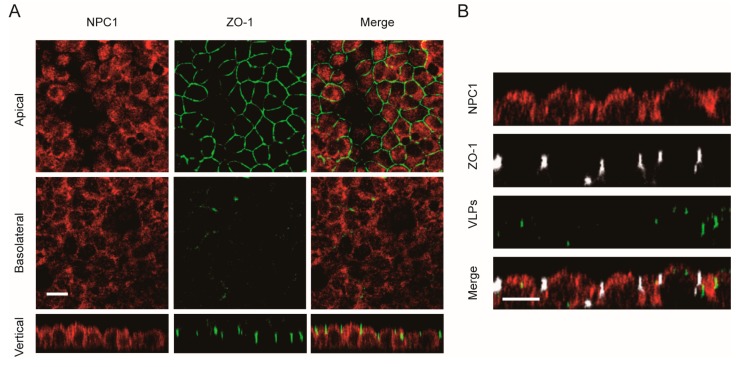
Nonpolarized distribution of the NPC1 receptor in polarized Vero cells. (**A**) NPC1 staining was detected in both apical and basolateral sections. Scale bar: 10 μm. (**B**) Confocal images representing the distribution of the NPC1 receptor during EGFP-VLP internalization into polarized Vero cells. Scale bar: 10 μm.

The distribution of the intracellular receptor NPC1 was also examined in polarized cells. As shown in Figure 6A, the NPC1 receptor was observed to be uniformly distributed in polarized Vero cells. After the cells were incubated with Ebola VLPs, there was no significant change in the NPC1 distribution (Figure 6B). Thus, the NPC1 receptor has a nonpolarized distribution, which suggests that it may not be responsible for the polarized entry of EBOV in polarized cells.

## 4. Discussion

Although many studies have been carried out to study EBOV infection, few studies have been dedicated to elucidating the virus infection mechanism in polarized cells. In this study, we report that EBOV prefers to enter polarized Vero cells from the apical cell surface. The receptors TIM-1 and Axl, which were distributed in an apically polarized manner, were found to be involved in the efficient apical entry of EBOV virus. 

After the polarization of epithelial and endothelial cells is established, some proteins and receptors are selectively expressed and transported to the apical or basolateral membrane [32]. To obtain access to polarized host cells, viruses must overcome the epithelial barrier to initiate and establish infection, which involves different strategies [33]. For example, chikungunya virus and hantavirus interact with receptors to infect epithelial cells from the apical domain [33,34]. However, other viruses, such as Crimean–Congo hemorrhagic fever virus and human cytomegalovirus, can utilize basolateral entry receptors for initial infection [35,36]. Finally, similar to hepatitis C virus and Nipah virus, some viruses can gain entry from either apical or basolateral membranes [25,37]. Here, our results show that EBOV enters polarized Vero cells from the apical cell surface. This finding might provide insights to explain how the virus crosses the polarized epithelial cell barrier to invade the host. 

Viruses often utilize host transport pathways to enter the cells. Macropinocytosis is the main pathway for Ebola virus entry [38,39]. Our results showed that macropinocytosis had no polarization characteristic, which seems not to be responsible for mediating the apical entry of the virus. Ebola GP mediates virus entry through receptor binding, and GP protein in virus-like particles (VLPs) affects epithelial and endothelial barrier functions [3,6,40]. TIM-1 is critical for Ebola entry through direct binding with phosphatidylserine (PtdSer) on the viral envelope, leading to virus internalization [41]. In addition to TIM-1, another related PtdSer-binding receptor, Axl, has been shown to promote the infection of several different enveloped viruses in a manner independent of specific receptor recognition of viral envelope glycoproteins [23,42,43]. Our study showed that TIM-1 and Axl had a preferential apical distribution pattern in polarized Vero cells. The preferential apical infection was related to the distribution of the TIM-1 and Axl receptors, and blocking the interaction between VLPs and the receptors reduced the apical infection efficiency. Our work also indicated that the intracellular receptor NPC1 did not show a polarized distribution, and it seems not to be responsible for the apical virus entry. Recently, it has been reported that selective expression of heparan sulfate on the basolateral surface of polarized Caco-2 cells can impart increased basolateral entry of EBOV [44]. In our study, heparan sulfate was weakly expressed and showed no polarized distribution in basolateral domains in polarized Vero C1008 cells (Figure S7). Thus, in polarized Vero C1008 cells, the TIM-1 and Axl receptors, but not NPC1 or heparan sulfate, are important factors for EBOV apical infection. These results further demonstrate that differential localization of viral receptors on the membrane can lead to polarized viral entry.

In summary, we revealed that EBOV prefers to enter polarized Vero cells from the apical cell surface. Viral receptors TIM-1 and Axl are distributed in an apically polarized manner in polarized cells, and their polarized localization leads to apical viral entry. Moreover, this study established a successful model for investigating EBOV infection. The polarized viral entry and its mechanism will help in further understanding EBOV infection and pathogenesis. 

## Supplementary Materials

The following are available online at https://www.mdpi.com/1999-4915/11/12/1117/s1: Figure S1: The effective removal of cell surface-attached VLPs; Figure S2: Construction of Ebola VLPs labeled with EGFP; Figure S3: The flow cytometry data; Figure S4: EBOV EGFP-VLPs could enter nonpolarized cells equally from every membrane; Figure S5: The macropinosomes appeared evenly distributed in the polarized Vero C1008 cells; Figure S6: Distribution of TIM-1 or Axl in nonpolarized Vero cells; Figure S7: The distribution of heparan sulfate in polarized Vero cells.
