# Supplementary figures and images for: Ebola Virus Uptake into Polarized Cells from the Apical Surface
Source: Viruses. 2019 Dec 2;11(12):1117. doi: 10.3390/v11121117 (PMC6949903; doi:10.3390/v11121117)

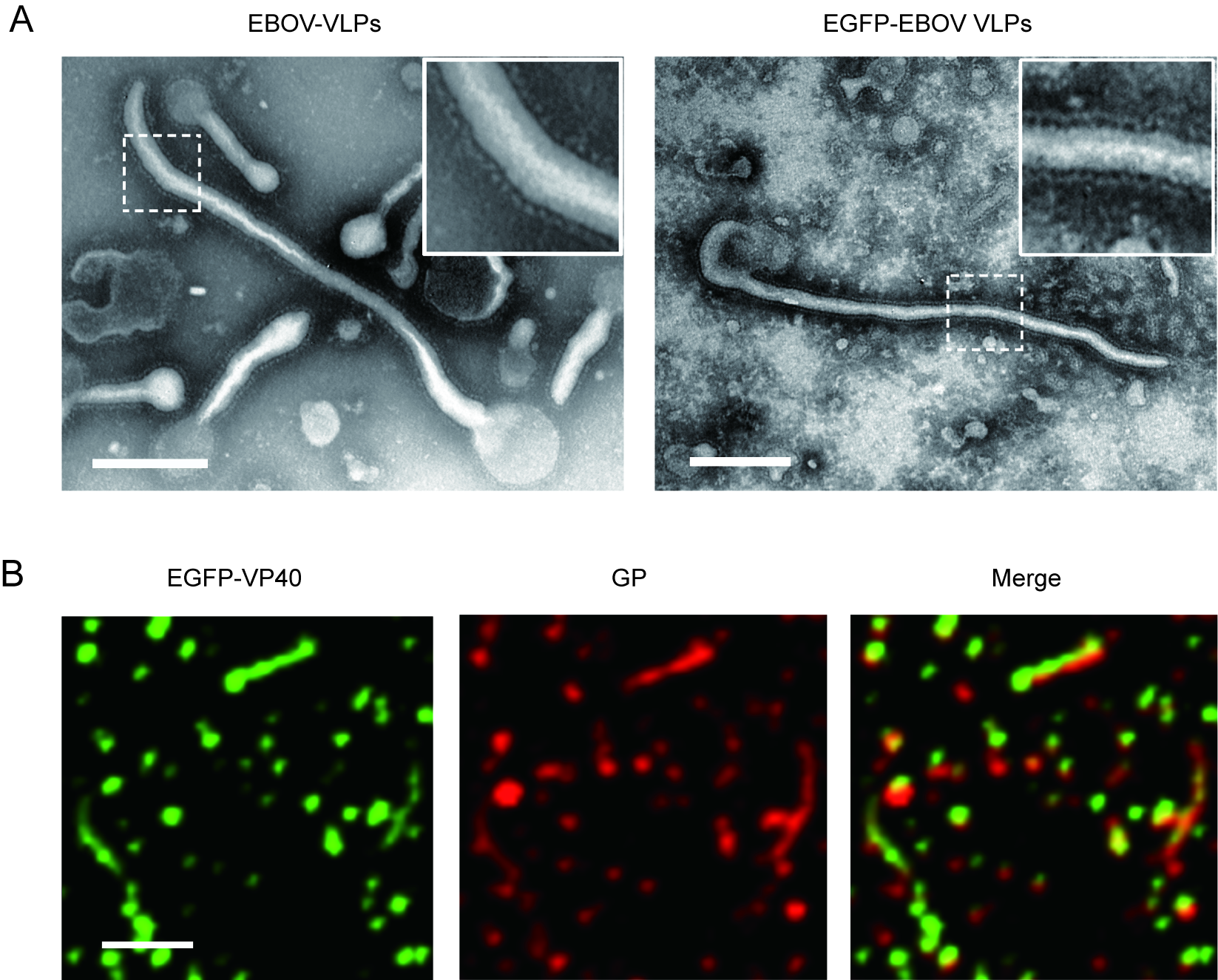

Supplement: Supplementary file 1 [file viruses-11-01117-s001.zip › viruses-638804-supplementary/[Viruses] Figures/Fig-1.tif]

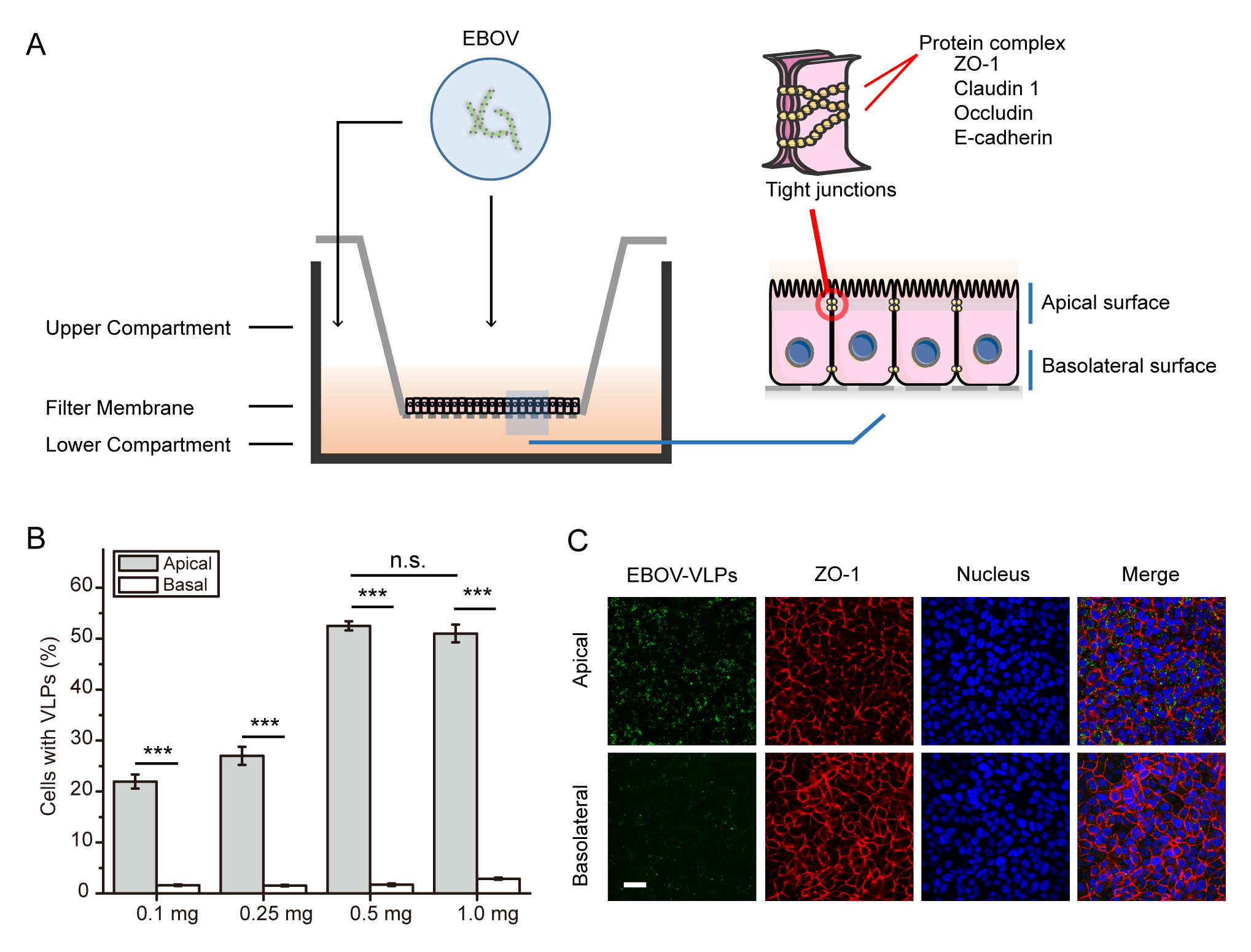

Supplement: Supplementary file 1 [file viruses-11-01117-s001.zip › viruses-638804-supplementary/[Viruses] Figures/Fig-2.tif]

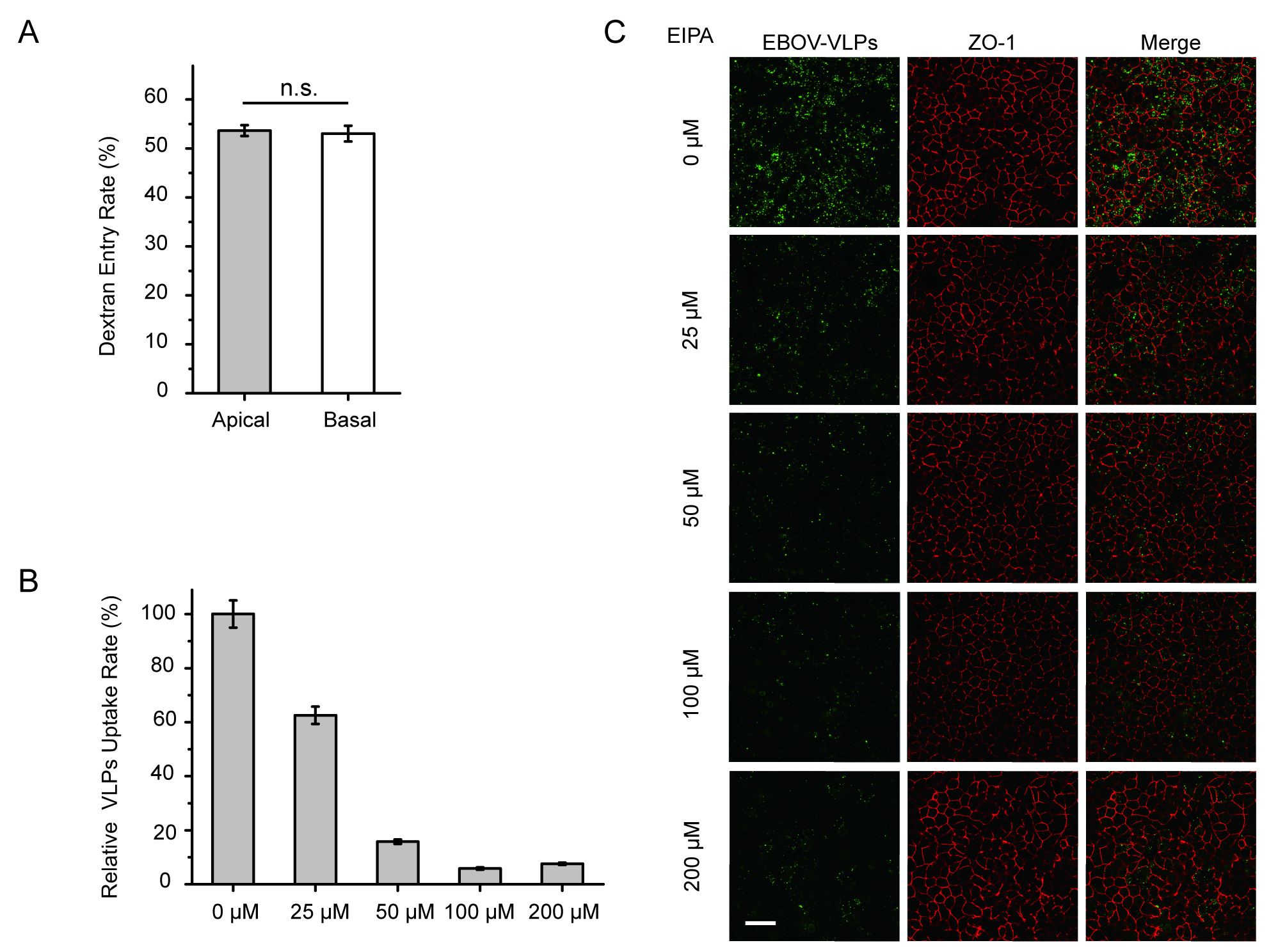

Supplement: Supplementary file 1 [file viruses-11-01117-s001.zip › viruses-638804-supplementary/[Viruses] Figures/Fig-3.tif]

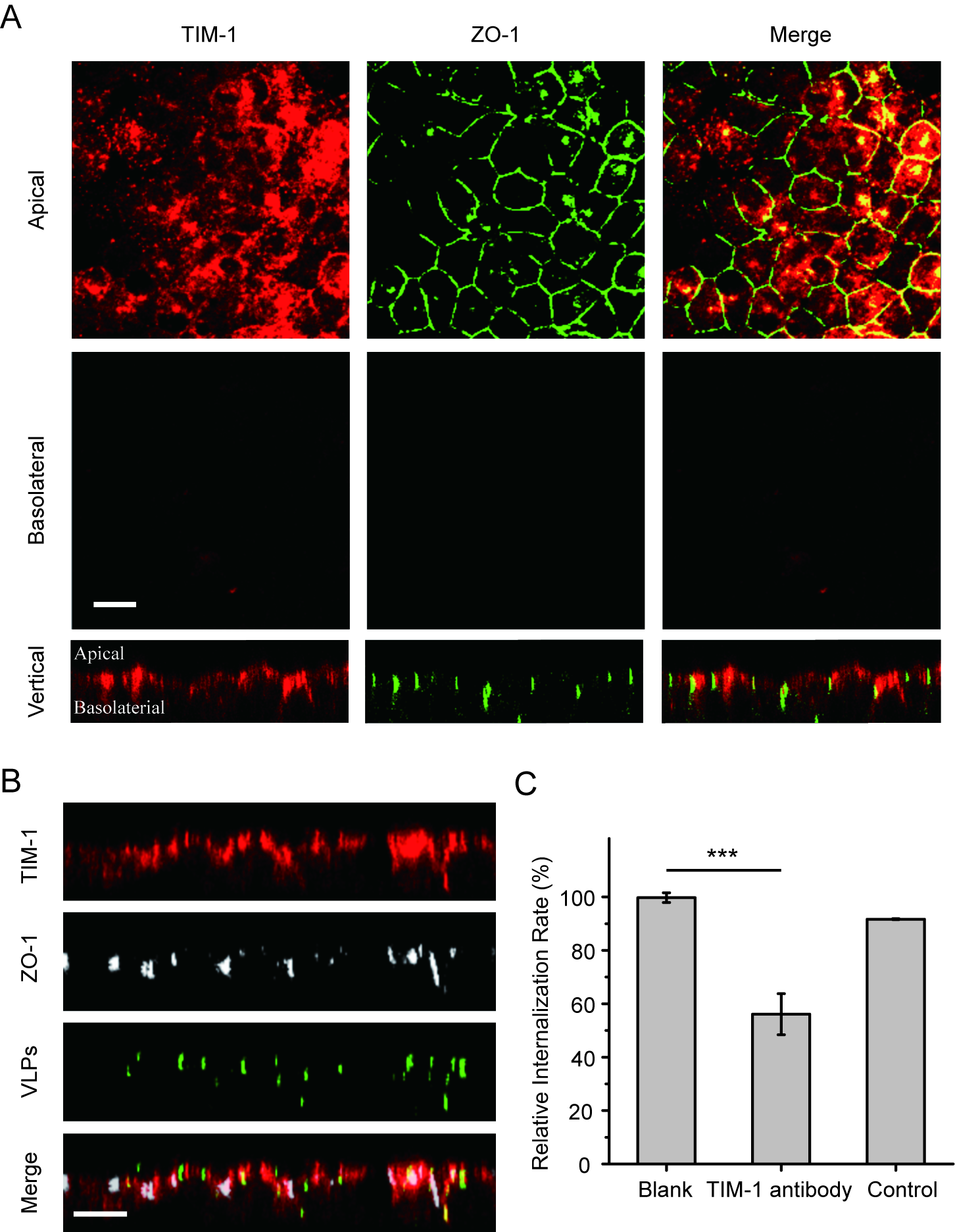

Supplement: Supplementary file 1 [file viruses-11-01117-s001.zip › viruses-638804-supplementary/[Viruses] Figures/Fig-4.tif]

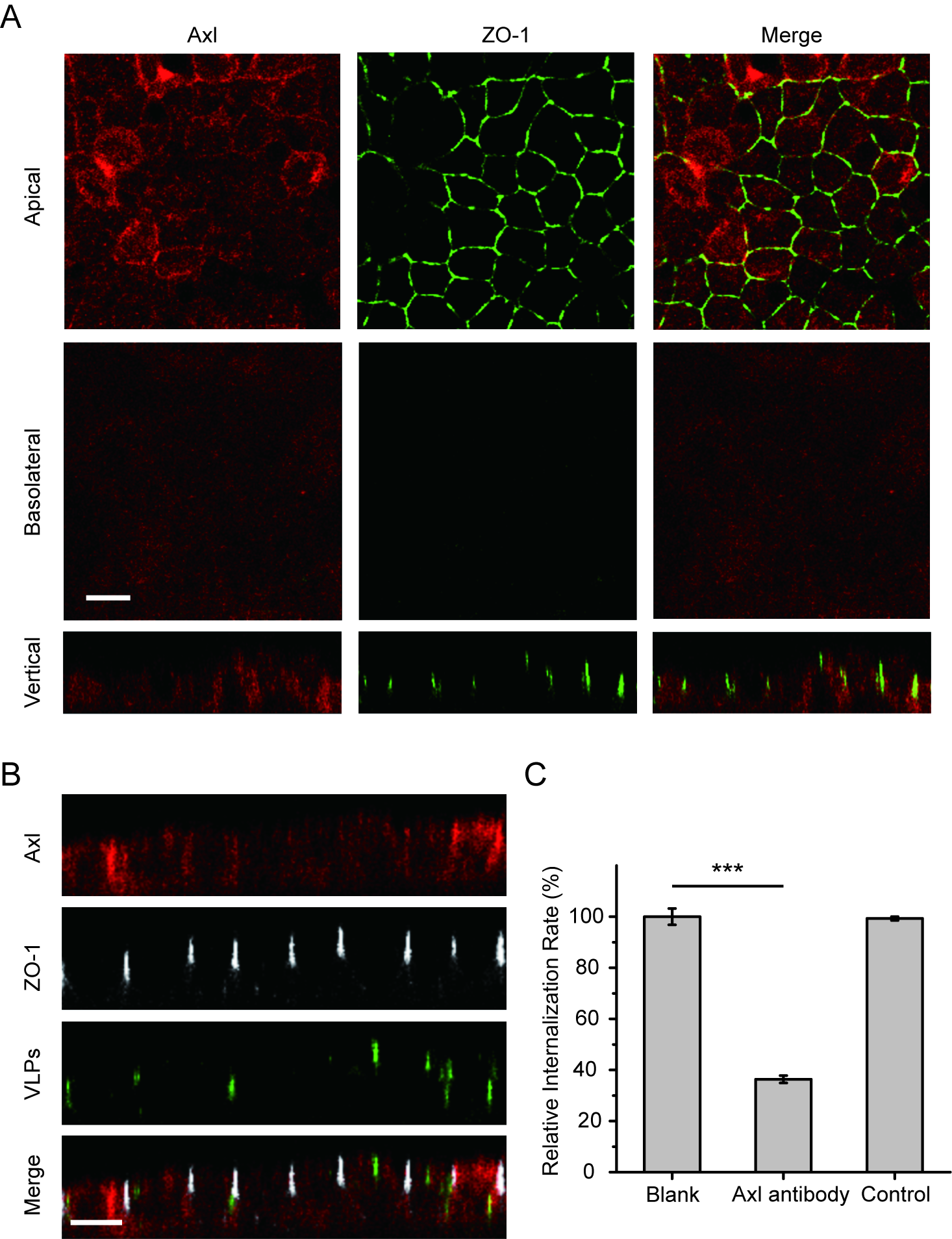

Supplement: Supplementary file 1 [file viruses-11-01117-s001.zip › viruses-638804-supplementary/[Viruses] Figures/Fig-5.tif]

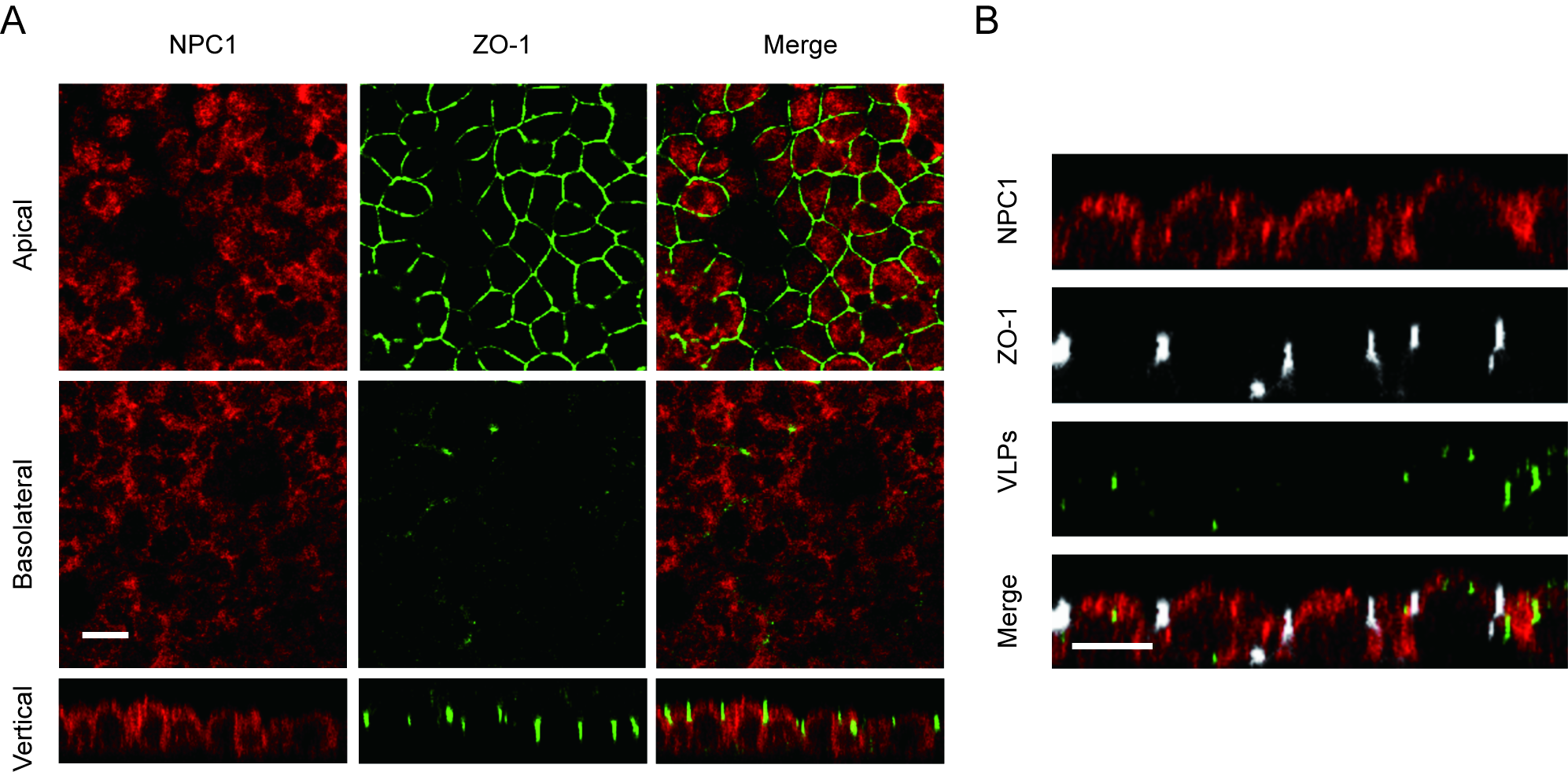

Supplement: Supplementary file 1 [file viruses-11-01117-s001.zip › viruses-638804-supplementary/[Viruses] Figures/Fig-6.tif]

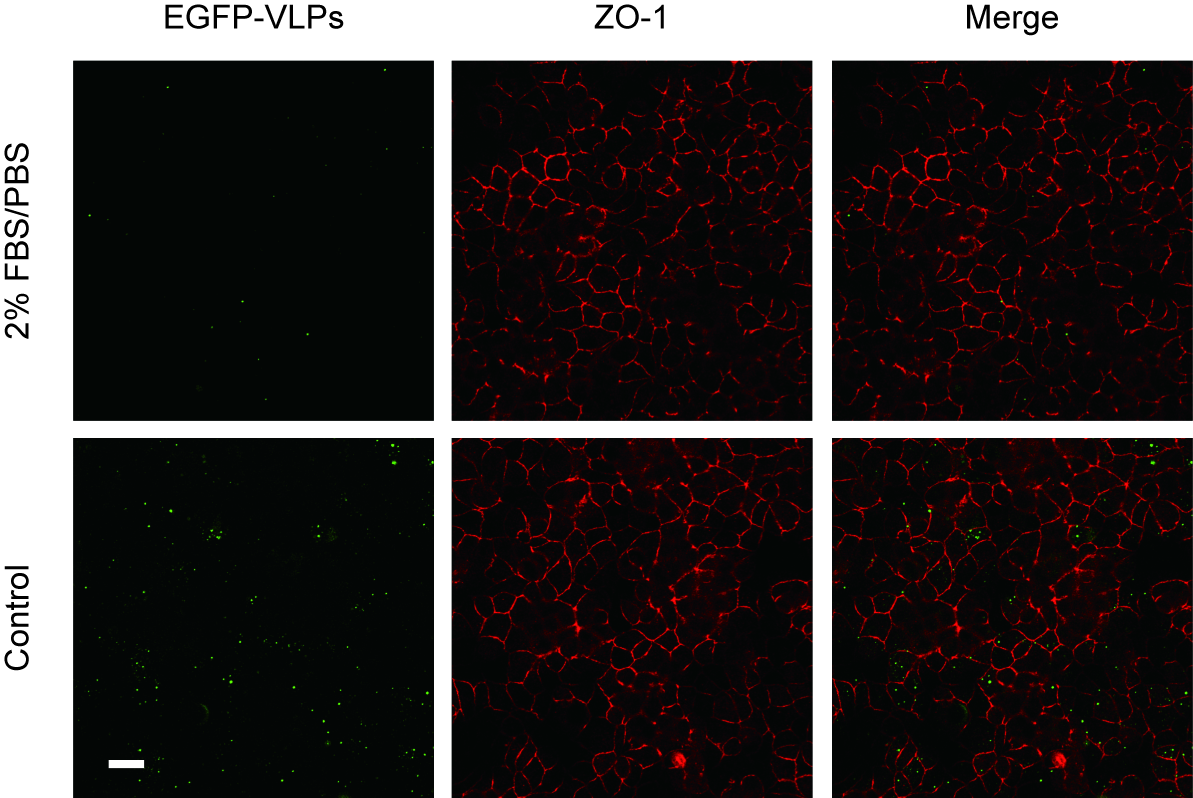

Supplement: Supplementary file 1 [file viruses-11-01117-s001.zip › viruses-638804-supplementary/[Viruses] Figures/Supplemental Figures/Fig-S1.tif]

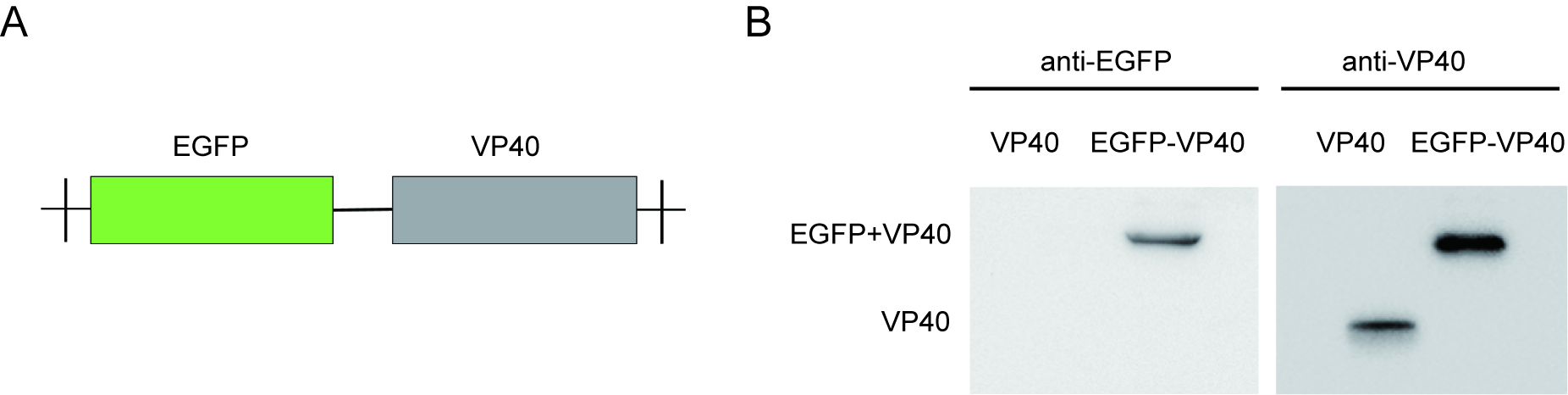

Supplement: Supplementary file 1 [file viruses-11-01117-s001.zip › viruses-638804-supplementary/[Viruses] Figures/Supplemental Figures/Fig-S2.tif]

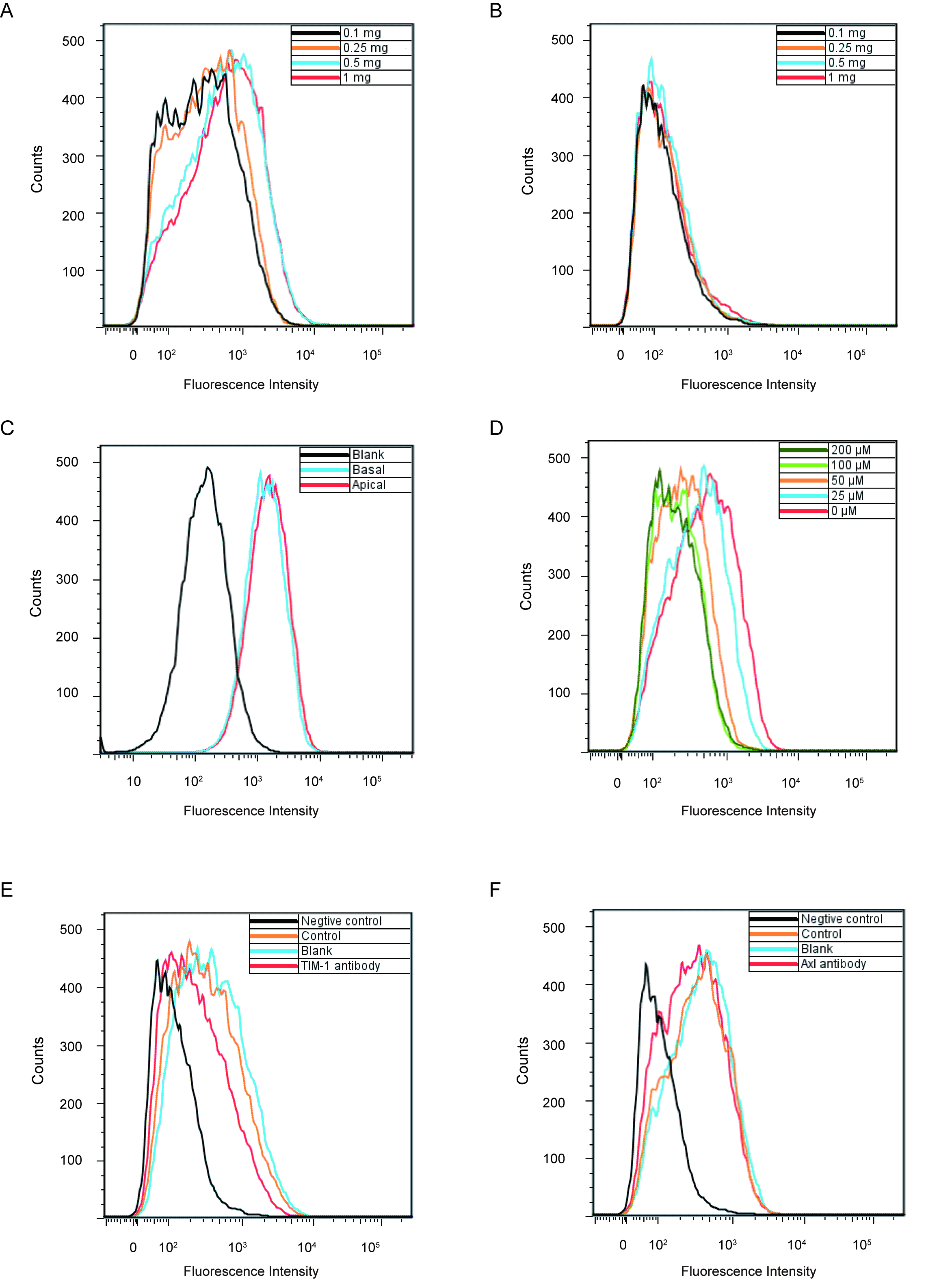

Supplement: Supplementary file 1 [file viruses-11-01117-s001.zip › viruses-638804-supplementary/[Viruses] Figures/Supplemental Figures/Fig-S3.tif]

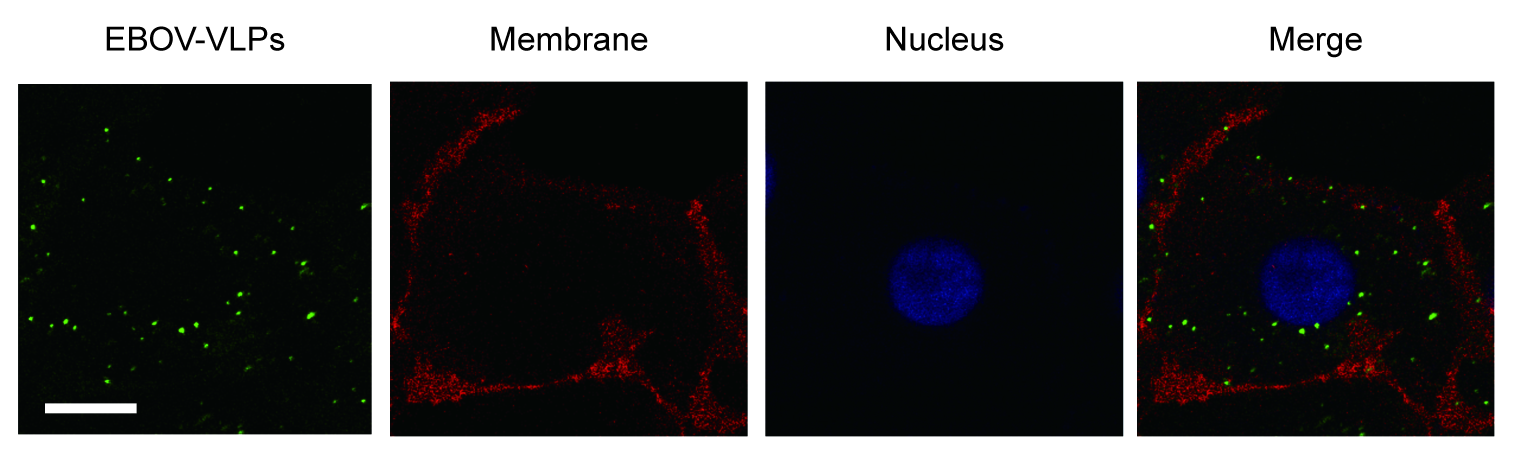

Supplement: Supplementary file 1 [file viruses-11-01117-s001.zip › viruses-638804-supplementary/[Viruses] Figures/Supplemental Figures/Fig-S4.tif]

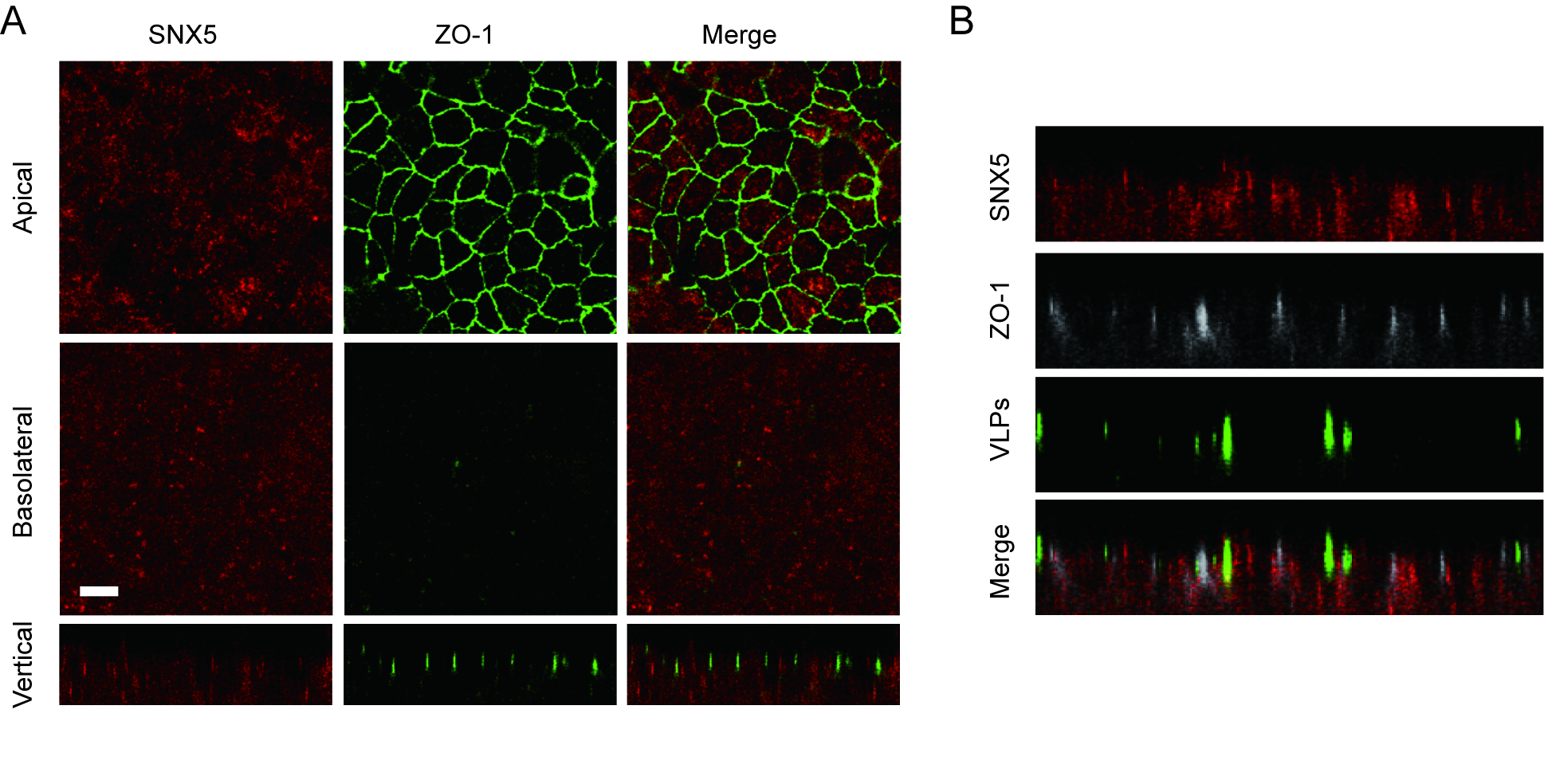

Supplement: Supplementary file 1 [file viruses-11-01117-s001.zip › viruses-638804-supplementary/[Viruses] Figures/Supplemental Figures/Fig-S5.tif]

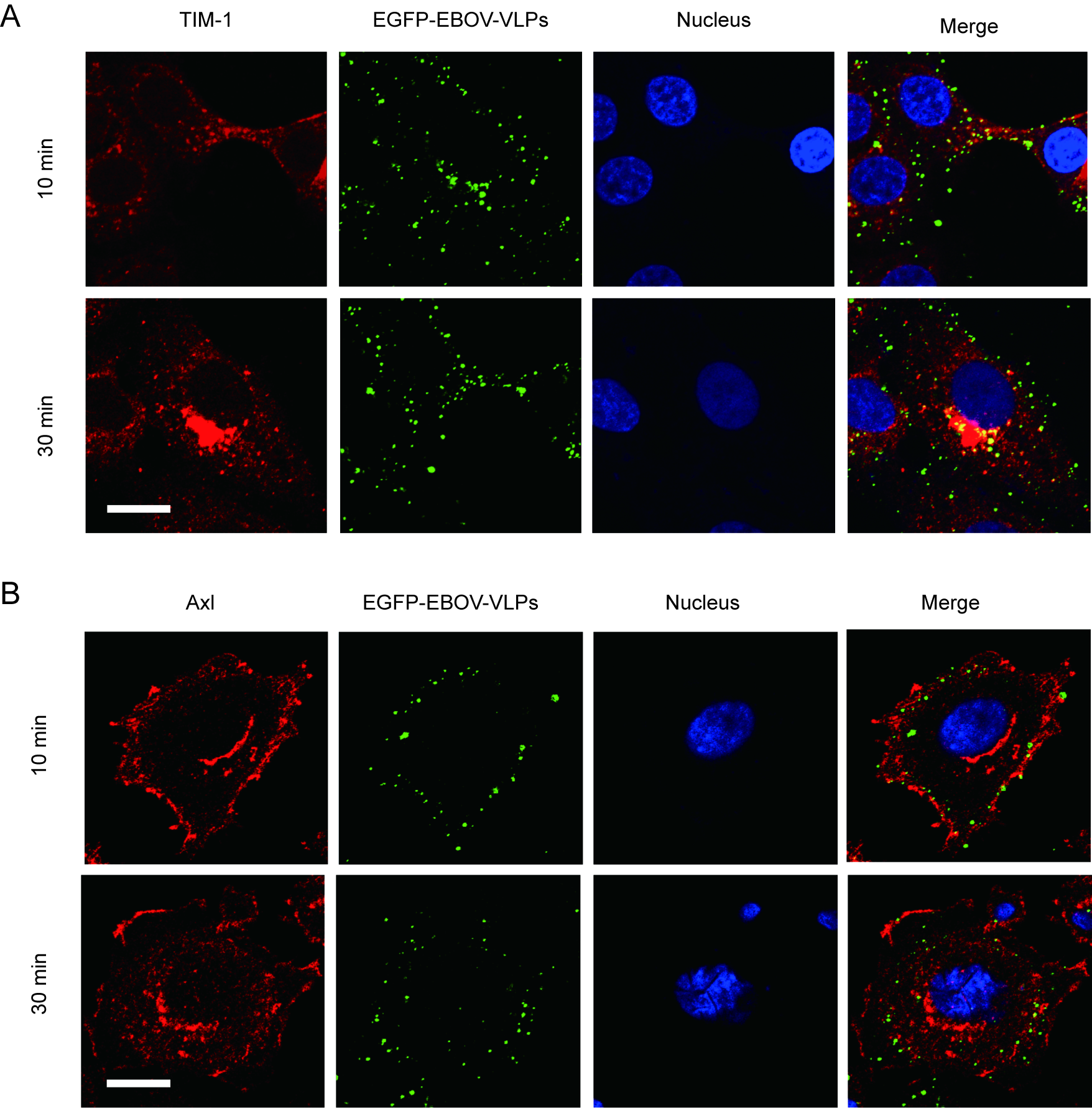

Supplement: Supplementary file 1 [file viruses-11-01117-s001.zip › viruses-638804-supplementary/[Viruses] Figures/Supplemental Figures/Fig-S6.tif]

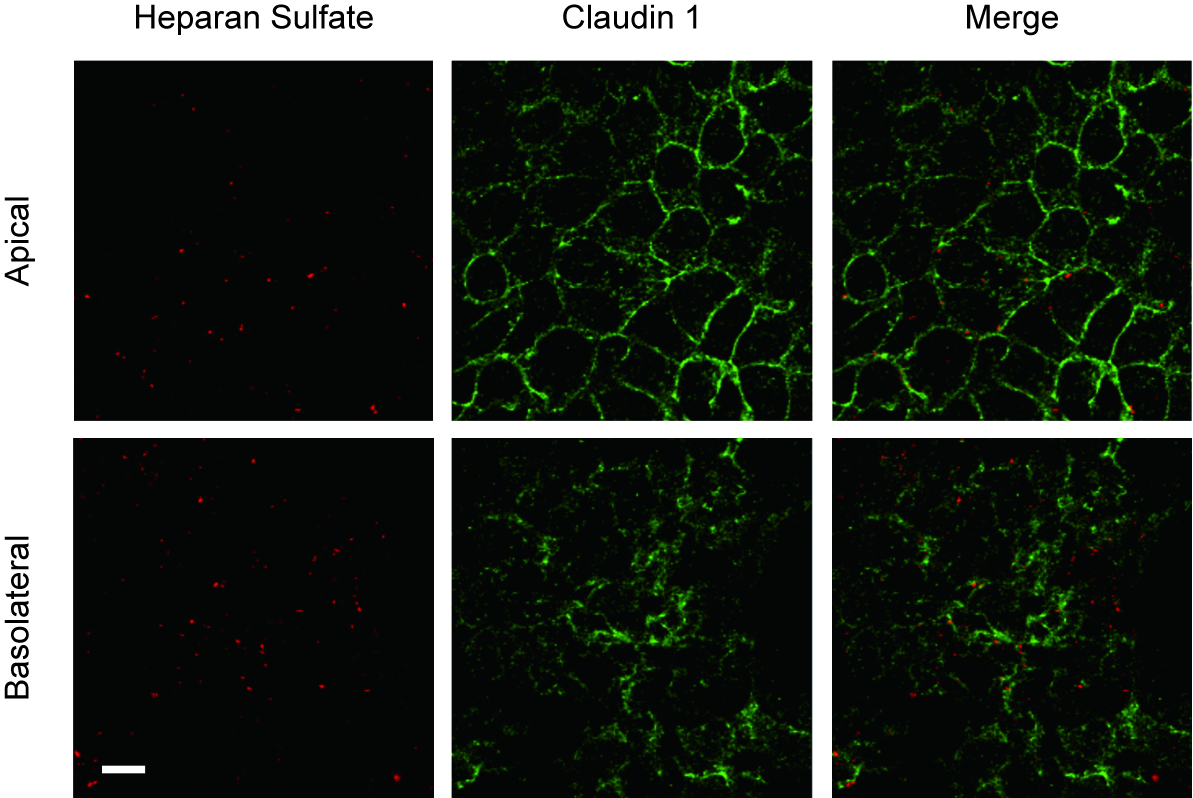

Supplement: Supplementary file 1 [file viruses-11-01117-s001.zip › viruses-638804-supplementary/[Viruses] Figures/Supplemental Figures/Fig-S7.tif]
